# Dynamic Alterations of Gut Microbiota in Porcine Circovirus Type 3-Infected Piglets

**DOI:** 10.3389/fmicb.2020.01360

**Published:** 2020-06-30

**Authors:** Lei Hou, Jing Wang, Wei Zhang, Rong Quan, Dan Wang, Shanshan Zhu, Haijun Jiang, Li Wei, Jue Liu

**Affiliations:** ^1^Beijing Key Laboratory for Prevention and Control of Infectious Diseases in Livestock and Poultry, Institute of Animal Husbandry and Veterinary Medicine, Beijing Academy of Agriculture and Forestry Sciences, Beijing, China; ^2^College of Veterinary Medicine, Yangzhou University, Yangzou, China; ^3^Jiangsu Co-innovation Center for Prevention and Control of Important Animal Infectious Diseases and Zoonoses, Yangzhou University, Yangzhou, China

**Keywords:** piglets, microbiota composition, dynamic changes, porcine circovirus type 3 infection, gut microbiota

## Abstract

Porcine circovirus type 3 (PCV3) is a novel porcine circovirus species associated with several diseases such as porcine dermatitis and nephropathy syndrome (PDNS)-like clinical signs, reproductive failure, cardiac pathologies, and multisystemic inflammation in piglets and sows. Currently, many studies have focused on the interaction between microbiota composition and disease progression. However, dynamic changes in the composition of the gut microbiota following PCV3 infection are still unknown. In this study, alterations in gut microbiota in PCV3-inoculated and sham-inoculated piglets were analyzed at various time points [7, 14, 21, and 28 days post-inoculation (dpi)] using the Illumina MiSeq platform. Using principal coordinate analysis, obvious structural segregations were observed in bacterial diversity and richness between PCV3- and sham-inoculated piglets, as well as at the four different time points. The abundance of gut microbiota exhibited a remarkable time-related decrease in *Clostridium_sensu_stricto_1* in PCV3-inoculated piglets. In addition, significant differences were observed in functional classification based on cluster of orthologous groups assignment, between PCV3- and sham-inoculated piglets. Our findings demonstrated that PCV3 infection caused dynamic changes in the gut microbiota community. Therefore, regulating gut microbiota community may be an effective approach for preventing PCV3 infection.

## Introduction

Porcine circoviruses (PCVs), belonging to the genus *Circovirus* within the Circoviridae family, are the smallest non-enveloped autonomously replicating DNA viruses with a single-stranded, circular genome (Rosario et al., 2017). Two genotypes of the porcine circovirus, porcine circovirus type1 (PCV1), and porcine circovirus type 2 (PCV2) have been extensively reported. Although PCV1 is considered a contaminant of cells and a non-pathogenic virus for pigs (Calsamiglia et al., 2002), PCV2 is recognized as an economically important pathogen that is associated with a diverse range of syndromes termed as porcine circovirus-associated (PCVAD), which includes reproductive failure, pneumonia, porcine dermatitis and nephropathy syndrome (PDNS), and post-weaning multisystemic wasting syndrome (PMWS) (Allan et al., 1998; Opriessnig et al., 2007; Afghah et al., 2017).

Recently, porcine circovirus type 3 (PCV3), an emerging species of the *Circovirus* genus, was first found in a case of PDNS and PMWS in the United States in 2015 (Phan et al., 2016; Palinski et al., 2017) and subsequently in Poland, Germany, Brazil, Italy, and China (Stadejek et al., 2017; Franzo et al., 2018; Fux et al., 2018; Shen et al., 2018; Tochetto et al., 2018; Wen et al., 2018). Retrospective studies have shown that PCV3 is highly homologous to bat-associated circovirus and that the first case of PCV3 infection occurred in 1966 in China (Fux et al., 2018). Recently, we experimentally inoculated a PCV3 strain obtained from infectious PCV3 DNA clone specific-pathogen-free (SPF) piglets aged 4 or 8 weeks and successfully reproduced PDNS-like disease (Jiang et al., 2019). Surprisingly, recent studies have shown that dogs and cattle could be infected with PCV3 (Zhang et al., 2018; Wang et al., 2019), indicating that PCV3 possesses an ability of cross-species transmission and circulation among a broad range of non-porcine hosts, which may pose a severe threat to the pig industry and to other animals as well.

Approximately, a trillion bacteria colonizing the mammalian gastrointestinal tract, collectively known as gut microbiota (Chen et al., 2017), play critical roles in exerting biological effects on host health, such as participating in several physiological and pathological processes, regulating host metabolism and immunity (Sommer and Backhed, 2013). Interestingly, growing evidences have shown that the alterations in gut microbiota is associated with progression of some diseases (McKenney and Pamer, 2015; Niederwerder, 2017; Tripathi et al., 2018). Moreover, age also plays a critical role in shaping gut microbes (Shibata et al., 2000). Therefore, it is necessary to explore the relationship between diverse growth stages in pigs and dynamic changes in the components of gut microbiota.

## Materials and Methods

### Ethics Statement

All the animal experiments were conducted in strict accordance with the animal welfare guidelines provided by the Institutional Animal Care and Use Committee (IACUC) Institute of Animal Husbandry and Veterinary Medicine, Beijing Academy of Agriculture and Forestry Sciences. All the animals were anesthetized using xylazine hydrochloride, resulting in minimal suffering.

### Animals and Sample Collection

Eight 4-week-old SPF Duroc Large-White piglets from the Beijing Center for SPF Swine Breeding and Management, China, were randomly divided into two groups: PCV3- and sham-inoculated groups (four piglets in each group). Each group was individually housed in a room and fed sterile food and water. Prior to inoculation, all piglets were detected as negative for antigens to PCV1, PCV2, PCV3, porcine parvovirus (PPV), torque teno virus (TTV), Japanese encephalitis virus (JEV), classical swine fever (CSFV), pseudorabies virus (PRV), swine influenza virus (SIV), transmissible gastroenteritis virus (TGEV), porcine epidemic diarrhea virus (PEDV), porcine reproductive and respiratory syndrome virus (PRRSV), and antibodies to PCV2, PPV, CSFV, TGEV, PEDV, and PRRSV. The two groups were differently treated as follows: (1) PCV3-inoculated group was intranasally inoculated with 2 ml of 10^6.53^ 50% tissue culture infective dose (TCID_50_)/ml of PCV3 strain LY (Jiang et al., 2019); (2) Control group was intranasally sham-inoculated with 2 ml of sterile DMEM. Fresh fecal samples from each piglet in both the groups were directly and individually collected using sterilizing tubes at 7, 14, 21, and 28 days post-inoculation (dpi) when piglets excreted feces and were immediately frozen in liquid nitrogen and stored at −80°C.

## DNA Extraction

Total DNA was isolated from the fecal samples using a DNeasy Blood & Tissue Minikit (Qiagen, Germany) in accordance with the manufacturer’s protocols. The final DNA concentration and purity were detected using a NanoDrop 2000 UV spectrophotometer (Thermo Fisher Scientific, Wilmington, MA, United States).

## Polymerase Chain Reaction Amplification

The V3–V4 hypervariable region of the bacterial 16S rRNA gene was amplified with primers by PCR [20-μl reaction volumes: 0.4 μl of Fast Pfu Polymerase, 0.8 μl of each primer (5 μM), 2 μl of 2.5 mM dNTPs, 4 μl of 5× Fast Pfu Buffer, and 10 ng of template DNA. The PCR program was set as follows: 3 min at 95°C, followed by 27 cycles (30 s at 95°C, 30 s at 55°C, and 45 s at 72°C], and a final extension at 10 min at 72°C using primers 338F (5′-ACT CCT ACG GGA GGC AGC AG-3′) and 806R (5′-GGA CTA CHV GGG TWT CTA AT-3′). Amplicons were confirmed using a 2% agarose gel, purified employing the AxyPrep DNA Gel Extraction Kit (Axygen Biosciences, Union City, CA, United States) and quantified by QuantiFluor^TM^-ST (Promega, Madison, WI, United States).

### Sequencing and Processing of Sequenced Data

Amplicons were pooled in equimolar and paired-end sequenced (2 × 300 bp) on an Illumina Miseq platform (Illumina, San Diego, CA, United States) according to the standard protocols (Lin et al., 2018) of Majorbio Bio-Pharm Technology Co., Ltd. (Shanghai, China). The sequencing data are deposited in the NCBI Sequence Read Archive (SRA) database (accession number: SRP249649). The 16S rRNA gene sequence information contained in this article was deposited in the GenBank Sequence Read Archive database under the accession number SRP249649.

Raw fastq files were demultiplexed, quality-filtered by Trimmomatic, and merged by FLASH using the following criteria: the reads were truncated at any site receiving an average quality score less than 20 over a 50-bp sliding window. The primers were matched exactly, allowing only two nucleotide mismatches, and the reads containing ambiguous bases were removed; sequences whose overlaps were longer than 10 bp were merged in accordance with their overlapping sequences.

In addition, the chimeric sequences were identified and removed by UCHIME,^1^ and were classified as the same operational taxonomic units (OTUs) according to 97% similarity using UPARSE version 7.1.^2^ Each 16S rRNA gene sequence was taxonomically assigned according to the rRNA sequences in the Silva database (SSU123), using the RDP Classifier algorithm^3^ with a confidence threshold of 70%.

### Quantification of PCV3 DNA by Real-Time PCR

To detect PCV3 DNA in serum samples, total DNA was isolated from each serum sample of the PCV3- and sham-inoculated piglets at 7, 14, 21, and 28 dpi using a DNeasy Blood & Tissue Minikit (Qiagen) in accordance with the manufacturer’s instructions. A 102-bp fragment of the PCV3 cap gene was amplified using the sense primer (5′-GTG CCA GGG CTT GTT ATT CT-3′) and antisense primer (5′-CTA TTC ATT AGG AGG CCC ACA G-3′) according to a real-time PCR protocol described recently (Jiang et al., 2019).

### Histopathology and Immunohistochemistry

The small intestines from PCV3- or sham-inoculated piglets collected at 28 dpi were formalin-fixed, paraffin-embedded, sectioned, and stained with hematoxylin and eosin (HE) for microscopic examination. To detect PCV3 viral antigen, the small intestines were subjected to immunohistochemistry (IHC) staining for histopathological observations. The histopathological lesions were mainly observed for lymphoid depletion, infiltration of inflammatory cells, and histiocytic hyperplasia. A polyclonal antibody against PCV3 was used for IHC, as described previously (Jiang et al., 2019).

### Statistical Analysis

Statistical comparisons of unweighted UniFrac distances among groups were performed via analysis of similarities (ANOSIM) using the “vegan” package of R (v3.0.3). *P* < 0.05 was considered significant. Statistical comparisons of alpha diversity were performed using Student’s *t*-test.^4^ Heatmaps were generated with the R-package gplots at the genus level. clusters of orthologous groups (COG) category assignments were performed through BLAST-based similarity searches to identify the closest matching sequences in the STRING database (Search Tool for the retrieval of interacting genes^5^) (E-value < 10^–6^).

## Results

### Clinical Symptoms and Pathological Changes Induced by PCV3 Infection

None of the sham-inoculated piglets exhibited clinical symptoms or obvious macroscopic lesions at any time throughout the experiment. However, in the PCV3-inoculated piglets, moderate clinical symptoms, including anorexia, emaciation, and coughing, were observed in all the four piglets at 12 dpi, while more severe symptoms, such as shivering, enhanced respiratory rates, and multifocal papules on the skin, were observed in the four piglets after 12 dpi; some clinical signs persisted until the end of the animal experiment.

**FIGURE 1:**
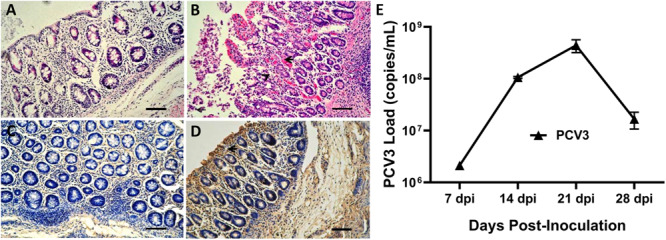
Histopathological lesions and immunohistochemical staining of small intestines and porcine circovirus type 3 (PCV3) loads in sera of PCV3- and sham-inoculated piglets. **(A)** Normal morphology of a small intestine section from a sham-inoculated piglet. **(B)** Small intestine lesions from a PCV3-inoculated piglet showed mucosal epithelial cell necrosis and some necrosis of lymphocytes, infiltration of abundant eosinophils and lymphocytes, and small numbers of plasma cells (arrowheads). **(C)** No staining was observed in the small intestine section from a sham-inoculated piglet. **(D)** Several PCV3 antigen-positive cells (arrowhead) were also observed in the small intestine of a PCV3-inoculated piglet, which were stained brown. Bars, 80 μm. **(E)** Viral loads in sera were quantitatively assayed by real-time PCR in PCV3-inoculated piglets. The values presented are the means of the results from the four PCV3-inoculated piglets at 7, 14, 21, and 28 dpi. Error bars indicate standard deviations.

None of the sham-inoculated piglets exhibited remarkable microscopic lesions in the small intestines (Figure 1A), while mild or severe gross lesions were observed in PCV3-inoculated piglets (Figure 1B). The small intestines exhibited mucosal epithelial cell necrosis and some lymphocyte necrosis, abundant eosinophils, macrophages, and a small amount of plasma cell infiltration. The blood vessels present under the mucosa of the small intestines were significantly dilated and congested. The small intestine samples of all the piglets were further subjected to IHC staining to detect the PCV3 antigen. As shown in Figure 1D, intestinal epithelial cells were positive for PCV3 antigen in the PCV3-inoculated piglets, while no positive signals were observed in sham-inoculated piglets (Figure 1C).

### Results of qPCR

Eight 4-week-old piglets were divided into two groups and intranasally inoculated with PCV3 and sterile DMEM, respectively. Serum samples were collected from each piglet in the two groups (eight samples) at 7, 14, 21, and 28 dpi and were assayed for PCV3 loads by absolute quantitative real-time PCR (qPCR). Negative results for PCV3 viremia in the four sham-inoculated piglets persisted till the end of animal experiment. The results of PCV3 loads for the PCV3-inoculated piglets at the above-indicated four time points after inoculation are shown (Figure 1E). The PCV3 genomic copy numbers in the serum samples ranged from 2.13 × 10^6^ to 4.98 × 10^8^ copies/ml for the PCV3-inoculated piglets, with peak levels at 21 dpi.

### Characteristics of Sequencing Data

The PCR products for the 16sRNA were qualified and were used for subsequent analysis (Supplementary Figure S1A). A total of 1,809,937 filtered sequences with an average length of 437 bp were collected across all the samples. The mean number of reads per sample was 56,561, ranging from 34,779 to 74,983. Good’s coverage for the obtained OTUs ranged from 99.64 to 99.85%, indicating that the sequencing results were accurate and reliable. The rarefaction curves were measured using Simpson, Shannon, Chao, and Sobs metrics and showed clear asymptotes, which confirmed adequate coverage range for all the samples (Supplementary Figures S1B–E).

**FIGURE 2:**
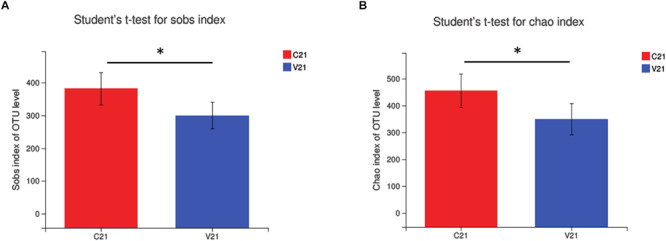
Changes in alpha diversity of gut microbiota in PCV3- (V) and sham-inoculated (C) piglets were analyzed using **(A)** Sobs index and **(B)** Chao index. Statistical differences at 21 days after PCV3 inoculation were calculated. Values are expressed as mean ± SD, with four piglets per group. ^∗^*P* < 0.05.

To investigate the patterns and levels of diversity in PCV3- (V) and sham-inoculated piglets (C), alpha diversity was measured. Sobs and Chao indices, representing the bacterial diversity and richness, exhibited a significant decrease only at 21 dpi in the PCV3-inoculated piglets (*P* < 0.05), while no difference was observed at the other indicated time points in the PCV3- and sham-inoculated piglets (*P* > 0.05) (Supplementary Figure S2). Further analysis demonstrated that the Sobs and Chao indices only at 21 dpi in the PCV3-inoculated piglets were lower than those in sham-inoculated piglets (*P* < 0.05) (Figure 2), while no differences was exhibited at 7, 14, and 28 dpi between the PCV3- and sham-inoculated piglets (*P* > 0.05, data not shown).

**FIGURE 3:**
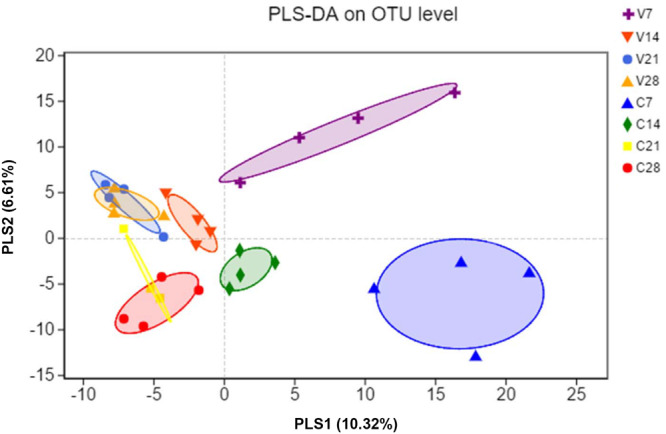
Partial least squares discriminant analysis (PLS-DA) was performed by analyzing the operational taxonomic unit (OTU) level of all samples. Color and shape represent the different groups. PLS-DA scatterplot shows obvious separation (*P* < 0.05, ANOSIM) and compositional differences in gut microbiota between PCV3- (V) and sham-inoculated (C) piglets at four time points (7, 14, 21, and 28 dpi); PLS1 and PLS2 represent 10.32 and 6.61% of variance, respectively (*X* and *Y*-axes).

To evaluate similarity of gut bacterial communities between PCV3- and sham-inoculated piglets, the OUT levels were calculated through partial least squares discriminant analysis (PLS-DA) based on unweighted UniFrac distances, a supervised analysis method. The bacterial communities between PCV3- and sham-inoculated piglets clustered separately, indicating that the overall composition of the bacterial communities in these two groups differed significantly (*P* < 0.05) (Figure 3). Moreover, further PLS-DA analysis showed distinct dynamic changes in PCV3- and sham-inoculated piglets (Figure 3).

#### Characterization and Dynamic Changes in Gut Microbiota in PCV3- and Sham-Inoculated Piglets

**FIGURE 4:**
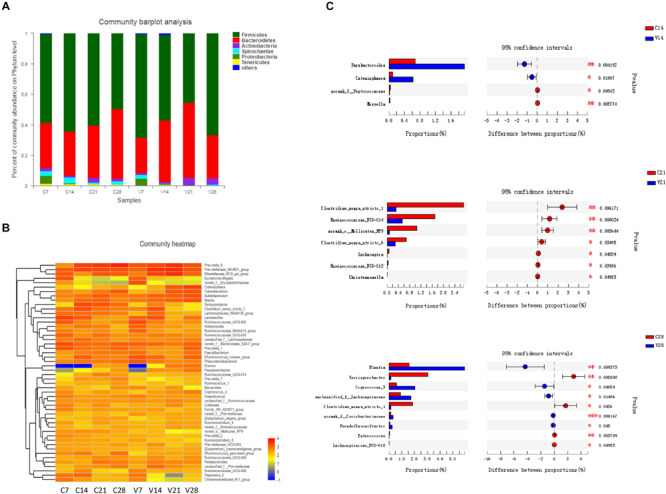
Relative abundance analysis of bacterial phyla and genera in PCV3- (V) and sham-inoculated (C) piglets. **(A)** Community bar-plot analysis represents relative abundance of sequences at the phylum level. **(B)** The heatmap depicts the percentage analysis of abundant genera in PCV3- (V) and sham-inoculated **(C)** piglets. The *X* and *Y*-axes represent the different groups and bacterial genera, respectively. Different color spots represent relative values (lg) of abundance. **(C)** The barplot illustrates the difference in predominant bacterial genera between PCV3- (V) from sham-inoculated (C) piglets at 14, 21, and 28 dpi. Statistical analysis was performed. Values are expressed as mean ± SD, with four piglets per group. *P < 0.05; **P < 0.01; and ***P < 0.001.

Fecal samples were collected from PCV3- and sham-inoculated piglets at four time points (7, 14, 21, and 28 dpi), and the dynamic changes in the gut microbiota were evaluated. The diversely dominant phyla among PCV3- and sham-inoculated piglets were identified by analyzing the percentage of community abundance. The bacterial communities of all the samples mainly contained six phyla, which were composed of more than 1% of the total sequences (Figure 4A). The dominant phyla at different time points in sham-inoculated piglets were *Firmicutes* (49.46–64.20%), *Bacteroidetes* (29.18–45.66%), *Actinobacteria* (1.01–3.04%), *Spirochaetae* (1.05–3.89%), *Proteobacteria* (0.12–5.07%), and *Tenericutes* (0.70–1.47%), whereas their abundance in PCV3-inoculated piglets was (45.39–68.12%), (23.43–49.32%), (1.02–4.69%), (0.13–2.54%), (0.20–4.23%), and (0.08–0.48%), respectively (Figure 4A). The abundance of *Actinobacteria* and *Tenericutes* was significantly increased and decreased at different time points in the PCV3-inoculated piglets (*P* < 0.01, *P* < 0.05, respectively), while no differences among the six phyla were observed in sham-inoculated piglets (*P* > 0.05) (Supplementary Figure S3A).

The differences in abundance in dominant genera were analyzed between PCV3- and sham-inoculated piglets. In the PCV3-inoculated group, *Prevotellaceae_NK3B31_group* (3.71–13.22%), *Prevotella_9* (5.66–13.21%), *Rikenellaceae_RC9_gut_group* (1.91–13.50%), *Catenisphaera* (0.08–8.86%), *Subdoligranulum* (2.29–5.32%), and *Blautia* (2.22–5.74%) were the dominant genera. The dynamic change in abundance in the genera mainly exhibited an increasing trend in *Subdoligranulum*, *Blautia*, *Catenisphaera*, and *Catenibacterium* from 7 to 28 dpi, while a significant decrease was observed in *Clostridium_sensu_stricto_1* (*P* < 0.05) (Supplementary Figure S3B). Meanwhile, in sham-inoculated piglets, the dominant genera were *Prevotella_9* (1.25–19.59%), *Prevotellaceae_NK3B31_group* (4.44–8.26%), *Rikenellaceae_RC9_gut_group* (1.16–5.41%), *Terrisporobacter* (0.15–6.95%), *norank_f_Bacteroidales_S24-7_group* (2.82–6.67%), and *Clostridium_sensu_stricto_1* (1.95–6.10%) (Figure 4B). We also observed a significant increase in the abundance of *Prevotella_9* (*P* < 0.05) and a corresponding decrease in *Lactobacillus* (*P* < 0.05) at four different time points of sample collection (Supplementary Figure S3B).

To further investigate the changes in gut microbiota, the abundance in genera was analyzed at diverse time points between PCV3- and sham-inoculated piglets (Figure 4C). No statistical difference was observed between the two groups at 7 dpi. A significantly higher abundance of *Parabacteroides* and *Catenisphaera* was observed in PCV3-infected group compared to that in sham-inoculated group at 14 dpi (*P* < 0.01, *P* < 0.05, respectively). At 21 dpi, the abundance of *Clostridium_sensu_stricto_1*, *Ruminococcaceae_UCG-014*, *Clostridium_sensu_stricto_6*, and *norank_o_Mollicutes_RF9* were significantly lower in PCV3-inoculated piglets than in sham-inoculated piglets (*P* < 0.01, *P* < 0.05). In addition, the abundance of *Blantia*, *Coprococcus_3*, and *unclassified_f_Lachnospiraceae* increased, and that of *Terrisporobacter* and *Clostridium_sensu_stricto_1* decreased in PCV3-inoculated piglets (*P* < 0.01, *P* < 0.05) at 28 dpi.

### Differences in the OTU-Level Phylogenetic Core of PCV3- and Sham-Inoculated Groups

Owing to significant difference in alpha diversity and peak levels of PCV3 loads at 21 dpi, the core microbiota was screened in accordance with more than 1% relative abundance in the OTU level and present at 21 dpi in the sham-inoculated piglets. Ten core microbiota of sham-inoculated piglets and the relative abundance of these genera in PCV3-inoculated piglets are shown in Table 1. *Prevotella_9* and *Rikenellaceae_RC9_gut_group* were predominant in the PCV3-inoculated group. Notably, the minimum relative abundance (0.24) of *Clostridium_sensu_stricto_1* was the only significant difference between the PCV3- and sham-inoculated group (*P* < 0.05) (Table 1).

**TABLE 1:** Comparisons of 10 core genera between porcine circovirus type 3 (PCV3)- (V) and sham-inoculated (C) piglets at 21 dpi.

**Phylum**	**Family**	**Genus**	**Mean relative**		***P*-values**
			**abundance (100%)**		
			**C**	**V**	
*Firmicutes*	*Clostridiaceae_1*	*Clostridium_sensu_stricto_1*	2.54	0.24	0.0108*
	*Lachnospiraceae*	*(Ruminococcus)_torques_group*	1.61	0.87	0.5047
	*Erysipelotrichaceae*	*Catenibacterium*	2.11	4.59	0.4424
	*Ruminococcaceae*	*Subdoligranulum*	1.88	3.17	0.3853
	*Acidaminococcaceae*	*Phascolarctobacterium*	2.00	1.27	0.0853
	*Peptostreptococcaceae*	*Terrisporobacter*	5.43	0.53	0.0857
	*Lactobacillaceae*	*Lactobacillus*	0.41	0.39	0.9655
*Bacteroidetes*	*Prevotellaceae*	*Prevotella_9*	3.92	9.51	0.2048
	*Rikenellaceae*	*Rikenellaceae_RC9_gut_group*	2.14	9.50	0.4311
	*Bacteroidales_S24-7_group*	*norank_f__Bacteroidales_S24-7_group*	0.97	0.96	0.9907

*P < 0.05.

### Functional Investigations and Comparisons Between PCV3- and Sham-Inoculated Piglets

**FIGURE 5:**
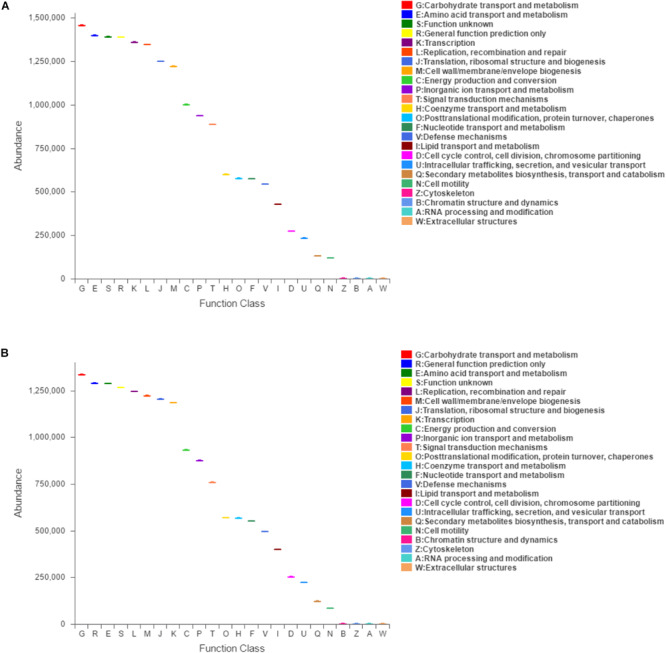
Clusters of orthologous groups (COG)-predicted functional classification at 21 dpi in PCV3- **(A)** and sham-inoculated piglets **(B)**. V and C letters denote PCV3- and sham-inoculated piglets, respectively. The *X* and *Y*-axes represent COG functional numbers and functional abundance. Boxes denote the interquartile range (IQR).

Function classification based on COG assignment was analyzed, and the relative abundances are further analyzed and described at 21 dpi in each category (Figure 5). Among these COG categories, “carbohydrate transport and metabolism” represented the richest function category in sham-inoculated piglets, followed by “amino acid transport and metabolism” and “function unknown” clusters. The largest function category in PCV3-inoculated piglets was classified as the “carbohydrate transport and metabolism,” followed by “general function prediction only” and “amino acid transport and metabolism.” The difference in function categories with COG parameters between PCV3- and sham-inoculated piglets is displayed in Table 2. In comparison with sham-inoculated piglets, the function categories of PCV3-inoculated piglets displayed obvious changes, such as “Cytoskeleton” and “Cell motility” (*P* < 0.01, *P* < 0.05, respectively).

**TABLE 2:** Comparisons of clusters of orthologous groups (COG) functional abundance between PCV3- (V) and sham-inoculated (C) piglets at 21 dpi.

**Function code**	**Family**	**Mean relative**		***P*-values**
		**abundance (100%)**		
		**C**	**V**	
G	Carbohydrate transport and metabolism	1,455,267	1,334,785	0.3823
E	Amino acid transport and metabolism	1,397,439	1,287,655	0.3562
S	Function unknown	1,389,336	1,266,500	0.3131
R	General function prediction only	1,388,125	1,289,089	0.3761
K	Transcription	1,358,973	1,184,231	0.2151
L	Replication, recombination, and repair	1,345,598	1,244,734	0.3036
J	Translation, ribosomal structure, and biogenesis	1,249,472	1,204,526	0.5874
M	Cell wall/membrane/envelope biogenesis	1,220,907	1,221,547	0.9929
C	Energy production and conversion	1,000,235	931,251	0.3798
P	Inorganic ion transport and metabolism	937,513	874,864	0.3279
T	Signal transduction mechanisms	887,192	758,306	0.1625
H	Coenzyme transport and metabolism	598,779	267,935	0.4376
O	Posttranslational modification, protein turnover, chaperones	575,670	568,515	0.3565
F	Nucleotide transport and metabolism	573,024	550,393	0.5382
V	Defense mechanisms	542,803	495,456	0.3713
I	Lipid transport and metabolism	427,994	398,736	0.2535
D	Cell cycle control, cell division, chromosome partitioning	272,507	251,723	0.3228
U	Intracellular trafficking, secretion, and vesicular transport	232,818	221,207	0.3652
Q	Secondary metabolites biosynthesis, transport and catabolism	130,313	120,593	0.1777
N	Cell motility	117,727	84,489	0.0342*
Z	Cytoskeleton	1,796	793	0.0008***
B	Chromatin structure and dynamics	1,632	1,202	0.2679
A	RNA processing and modification	112	91	0.6192
W	Extracellular structures	112	68	0.6906

*P < 0.05, ***P < 0.001.

## Discussion

In this study, PCV3 pathogenesis was assessed through pathological changes and viral loads. Similar histological lesions were observed between PCV3-inoculated piglets in our study and those previously reported in naturally PCV3-infected diseases (Jiang et al., 2019). Histopathological lesions in the small intestines were characterized by abundant lymphocyte necrosis and depletion, necrotic mucosal epithelial cells, and infiltration of inflammatory cells, including macrophages and eosinophils in PCV3-inoculated piglets. It is well known that eosinophils are a type of disease-fighting white blood cell that play an important role in inflammatory responses to allergic reactions, asthma, and infections (Patricia et al., 2013). Our further results showed abundant PCV3-positive cells in small intestine tissues of the PCV3-inoculated piglets, with epithelial cells exhibiting a positive reaction for the PCV3 antigen. PCV3 DNA loads were analyzed at various time points post-inoculation, and they were found to be similar to each other in the clinical phase of the disease in PCV3-inoculated piglets, with peak levels of viral DNA (approximately 4.98 × 10^8^ PCV3 genome copies/ml) at 21 dpi. In general, these data indicate that PCV3 possesses high replication capabilities and pathogenicity in virus-inoculated piglets.

Changes were observed in the composition of the gut microbiota after PCV3 infection, which contribute to a progressive understanding of the relationship between PCV3 infection and gut microbiota. Low diversity indicates an imbalance of gut microbiota, which is, therefore, vulnerable to disturbances and sensitivity to environmental factors (Chen et al., 2017). No statistical differences in the alpha diversity of gut microbiota were observed at all time points in the sham-inoculated piglets; however, a significantly decreasing trend of alpha diversity in the gut bacterial composition was observed in PCV3-inoculated piglets at 21 dpi (*P* < 0.05) compared to other inoculation time points. Meanwhile, PCV3-inoculated piglets showed an obvious decrease in alpha diversity compared to sham-inoculated piglets at 21 dpi (*P* < 0.05). The decreased alpha diversity in PCV3-inoculated piglets could have resulted in a stressed state of the body and, thus, could explain the increased morbidity and mortality.

A majority of gut microbiota belong to the *Firmicutes* and *Bacteroidetes* phyla (Nguyen et al., 2015; Huang et al., 2019), and the imbalance between the two has been implicated in many disorders and diseases (Clarke et al., 2012). Decreased *Firmicutes*/*Bacteroidetes* ratio is considered to be related to weight loss in obese individuals (Turnbaugh et al., 2006) and reduced capacity to obtain energy from diets (Ley et al., 2006). For example, elevated abundance of *Firmicutes* and reduced *Bacteroidetes* abundance were observed in obese mice (Ley et al., 2005) and in obese humans (Chakraborti, 2015). In our study, the abundance of *Firmicutes* and *Bacteroidetes* maintained relative stability in sham-inoculated piglets. Interestingly, PCV3 inoculation exhibited a decrease in the *Firmicutes* phylum from 7 to 21 dpi and an increase at 28 dpi, while an opposite trend was observed in the *Bacteroidetes* phylum. Importantly, the reduced *Firmicutes*/*Bacteroidetes* ratio in our study reflects the emaciation caused in the PCV3-inoculation piglets, which was similar to a previously reported decrease in average weekly weight gains in piglets inoculated with PCV3 (Jiang et al., 2019).

Some reports suggest that *Parabacteroides* and *Catenisphaera* contribute to inflammatory diseases, such as inflammatory bowel disease (IBD) (Kaakoush, 2015; Yang et al., 2019). In our study, *Parabacteroides* and *Catenisphaera* (members of the family *Erysipelotrichaceae*) were markedly increased (*P* < 0.01, *P* < 0.05, respectively) in PCV3-inoculated piglets at 14 dpi compared to sham-inoculated piglets (Kanno et al., 2015). Moreover, high levels of inflammatory cytokines were observed after PCV3 infection in a previous report (Jiang et al., 2019). Thus, these data suggest that *Parabacteroides* and *Catenisphaera* are involved in the production of inflammatory cytokines, which is one of the causes of inflammation responsible for PCV3-mediated tissue damage caused by the migration of inflammatory cells in PCV3-inoculated piglets. In addition, the relative abundance of three members of the phylum *Firmicutes* (*Clostridium_sensu_stricto_1*, *Ruminococcaceae_UCG-014*, and *Clostridium_sensu_stricto_6*) and *norank_o_Mollicutes_RF9* exhibited significantly lower levels (*P* < 0.01, *P* < 0.05, respectively) at 21 dpi in the PCV3-inoculated piglets compared to that in sham-inoculated piglets, which reduced the ratio of *Firmicutes* to *Bacteroidetes* phyla and finally weakened the capacity to obtain energy from diet, thereby, leading to weight loss (Ley et al., 2006; Henning et al., 2019; Jiang et al., 2019; Li et al., 2019). Moreover, *Blantia*, *Coprococcus*, and *unclassified_f_Lachnospiraceae* displayed positive anti-inflammatory effects, resulting in the weakening of inflammatory responses (Jenq et al., 2015; Di Iorio et al., 2019; Lee et al., 2020) and exhibiting a marked increase in PCV3-inoculated piglets at 28 dpi compared to those in sham-inoculated piglets (*P* < 0.01, *P* < 0.05). These results explain the downregulation of inflammatory cytokines, such as TNF-α or IL-6, in the later stage of PCV3 infection (Jiang et al., 2019). Meanwhile, *Terrisporobacter* and *Clostridium_sensu_stricto_1* from *Firmicutes* were significantly decreased at 28 dpi in PCV3-inoculated piglets (*P* < 0.01, *P* < 0.05, respectively), which further confirmed the weight loss of piglets after PCV3 infection.

The results showed that low diversity in gut microbiota and peak levels of PCV3 DNA are observed only at 21 dpi, indicating that PCV3 replication had the greatest influence on the gut microbiota, so our further experiments and analysis mainly focused on this time point. Notably, many microorganisms cooperate with bacteria to exert multiple functions in the intestinal microbial ecosystem (Huang et al., 2018). The intestines play an important role in preventing gut microbes from invading the host by building the first barrier. In the intestines, epithelial and endothelial cells are mainly involved in this resistance process to preserve tissue homeostasis (Zhang et al., 2015). Accordingly, alterations in the functions of such barriers are mostly attributed to gut inflammation. The cytoskeleton, a subcellular structure, is an important regulatory factor in maintaining cellular barriers (Lopez-Posadas et al., 2017). Some inflammatory cytokines can induce cytoskeletal rearrangements, causing inflammation-dependent defects in gut-barrier function (Mehta et al., 2015). In our study, *Parabacteroides* and *Catenisphaera* were responsible for the production of inflammatory cytokines, which decreased from the beginning of the 14 dpi in PCV3-inoculated piglets, partially explaining the relatively lower function abundance of “Cell motility” and “Cytoskeleton” caused by inflammatory injury. Taken together, these data indicate that PCV3 infection alters the diversity and composition of gut microbiota from the phylum to genus levels, ultimately disturbing normal physiological functions of the intestines.

## Conclusion

In conclusion, our results showed that PCV3 infection disrupted the gut microbiota community and mainly hampered dynamic changes in the abundances of *Parabacteroides*, *Catenisphaera*, *Clostridium_sensu_stricto_1*, *Ruminococcaceae_UCG-014*, *Clostridium_sensu_stricto_6*, and *norank_o_Mollicutes_RF9*, finally resulting in a severe inflammatory response and weight loss in the PCV3-inoculated piglets. Hence, these data will improve our understanding of the relationship between PCV3 infection and gut microbiota. They also indicate that the stability of the gut microbiota is a key factor in normal physiological conditions, which may be an effective strategy to defend against PCV3 infection.

## Data Availability Statement

The datasets generated for this study can be found in the https://www.ncbi.nlm.nih.gov/sra/; Accession number SRP249649.

## Ethics Statement

The animal study was reviewed and approved by the Institutional Animal Care and Use Committee (IACUC), Institute of Animal Husbandry and Veterinary Medicine, Beijing Academy of Agriculture and Forestry Sciences.

## Author Contributions

LH, LW, and JL conceived and designed the experiments. LH, JW, SZ, DW, and HJ performed the experiments. LH, WZ, and RQ analyzed the data and contributed analysis tools. LH and JL wrote the manuscript. All authors read and approved the final manuscript.

## Conflict of Interest

The authors declare that the research was conducted in the absence of any commercial or financial relationships that could be construed as a potential conflict of interest.

## Footnotes

1 http://drive5.com/usearch/manual/uchime_algo.html

2 http://drive5.com/uparse/

3 http://rdp.cme.msu.edu/

4 www.majorbio.com

5 http://string-db.org/

## Supplementary Material

FIGURE S1 The PCR products for the 16S rRNA from PCV3- (V) and sham-inoculated (C) piglets at 7, 14, 21, and 28 dpi were exhibited **(A)**; M denotes DNA Markers (DL2000), NC denotes negative control. Bacterial rarefaction curves of different metrics were used to evaluate sequence coverage for all samples of PCV3- (V) and sham-inoculated (C) piglets at 7, 14, 21, and 28 dpi, with four piglets per group. Simpson index **(B)**, Shannon index **(C)**, Chao index **(D)**, and Rarefaction index **(E)**.

FIGURE S2 Alpha diversity differences in PCV3- (V) and sham-inoculated (C) piglets were evaluated using **(A,C)** Sobs index and **(B,D)** Chao index, with four piglets per group. Statistical differences at 21 dpi were calculated. Values are expressed as mean ± SD, with four piglets per group. ^∗^*P* < 0.05.

FIGURE S3 The differences in predominant bacterial phyla and genera are illustrated by the Community barplot at 21 dpi in PCV3- (V) and sham-inoculated (C) piglets. Percentage denotes relative abundance of bacterial phylum **(A)** and genus **(B)** in each group, with four piglets per group. Statistical analysis was calculated. Values are expressed as mean ± SD, with four piglets per group. ^∗^*P* < 0.05, ^∗∗^*P* < 0.01.
