# Supplementary figures and images for: Dynamic Alterations of Gut Microbiota in Porcine Circovirus Type 3-Infected Piglets
Source: Front Microbiol. 2020 Jun 30;11:1360. doi: 10.3389/fmicb.2020.01360 (PMC7341976; doi:10.3389/fmicb.2020.01360)

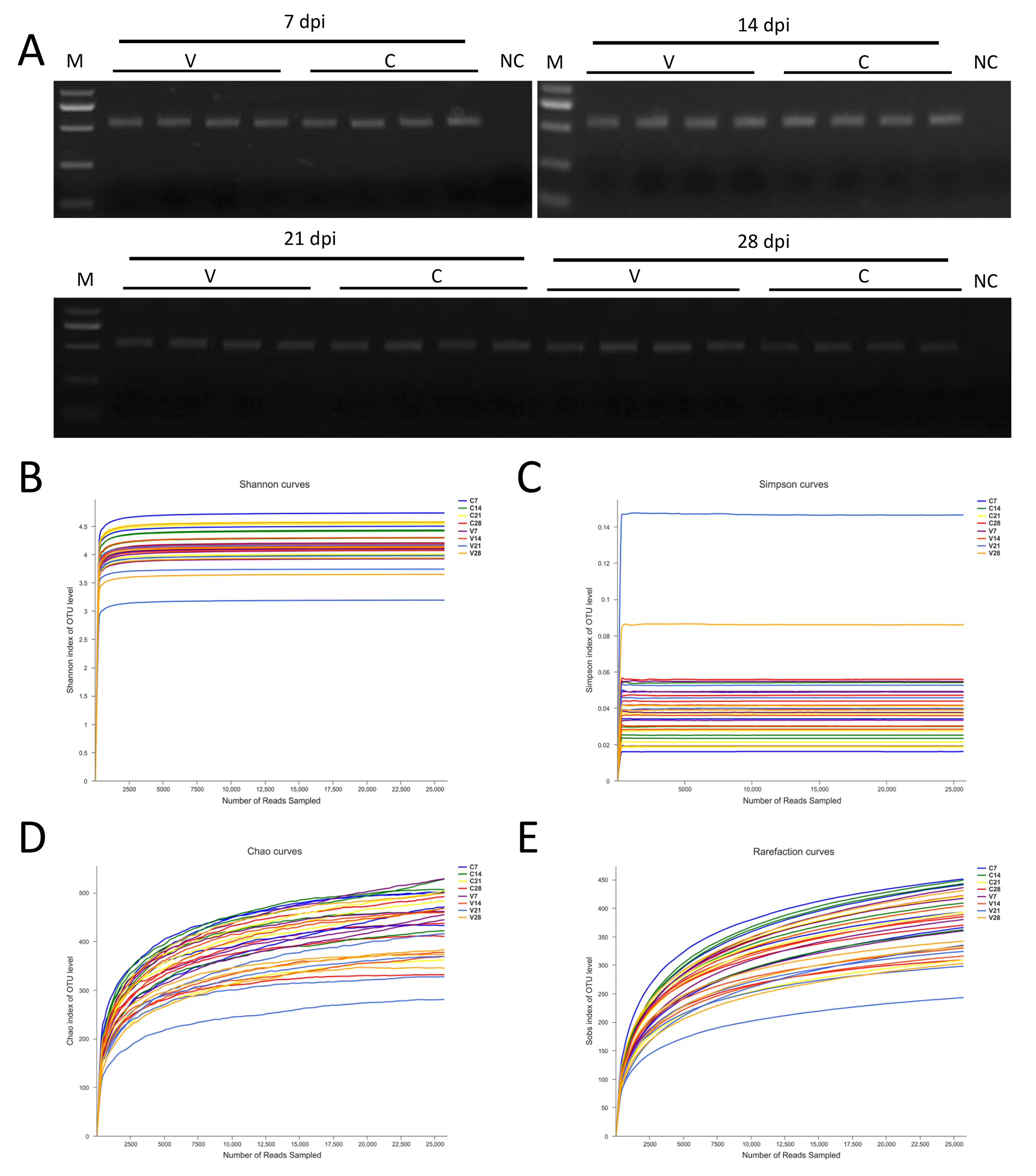

Supplement: FIGURE S1 — The PCR products for the 16S rRNA from PCV3- (V) and sham-inoculated (C) piglets at 7, 14, 21, and 28 dpi were exhibited (A); M denotes DNA Markers (DL2000), NC denotes negative control. Bacterial rarefaction curves of different metrics were used to evaluate sequence coverage for all samples of PCV3- (V) and sham-inoculated (C) piglets at 7, 14, 21, and 28 dpi, with four piglets per group. Simpson index (B), Shannon index (C), Chao index (D), and Rarefaction index (E). [file Image_1.TIF]

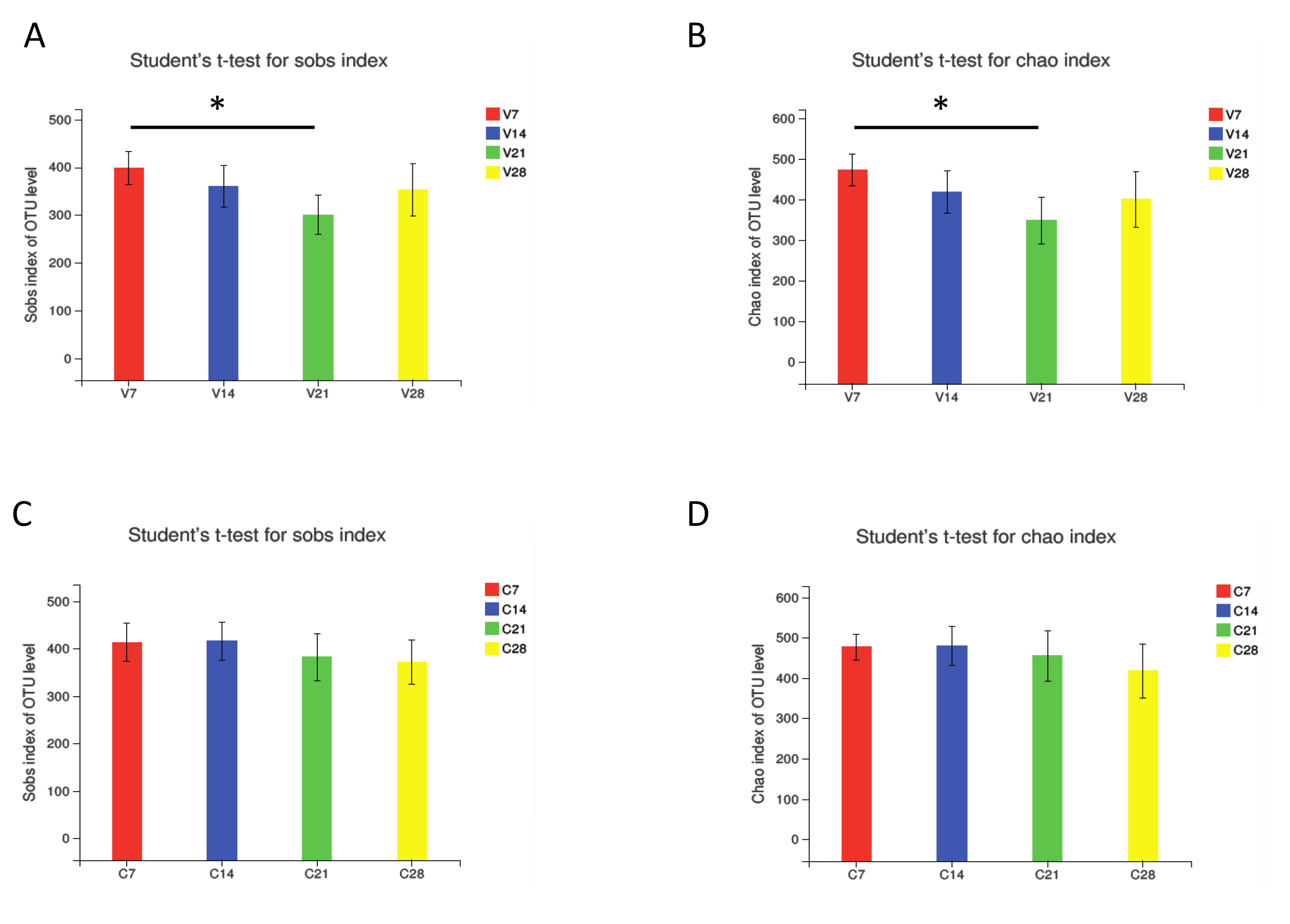

Supplement: FIGURE S2 — Alpha diversity differences in PCV3- (V) and sham-inoculated (C) piglets were evaluated using (A,C) Sobs index and (B,D) Chao index, with four piglets per group. Statistical differences at 21 dpi were calculated. Values are expressed as mean ± SD, with four piglets per group. ∗P < 0.05. [file Image_2.TIF]

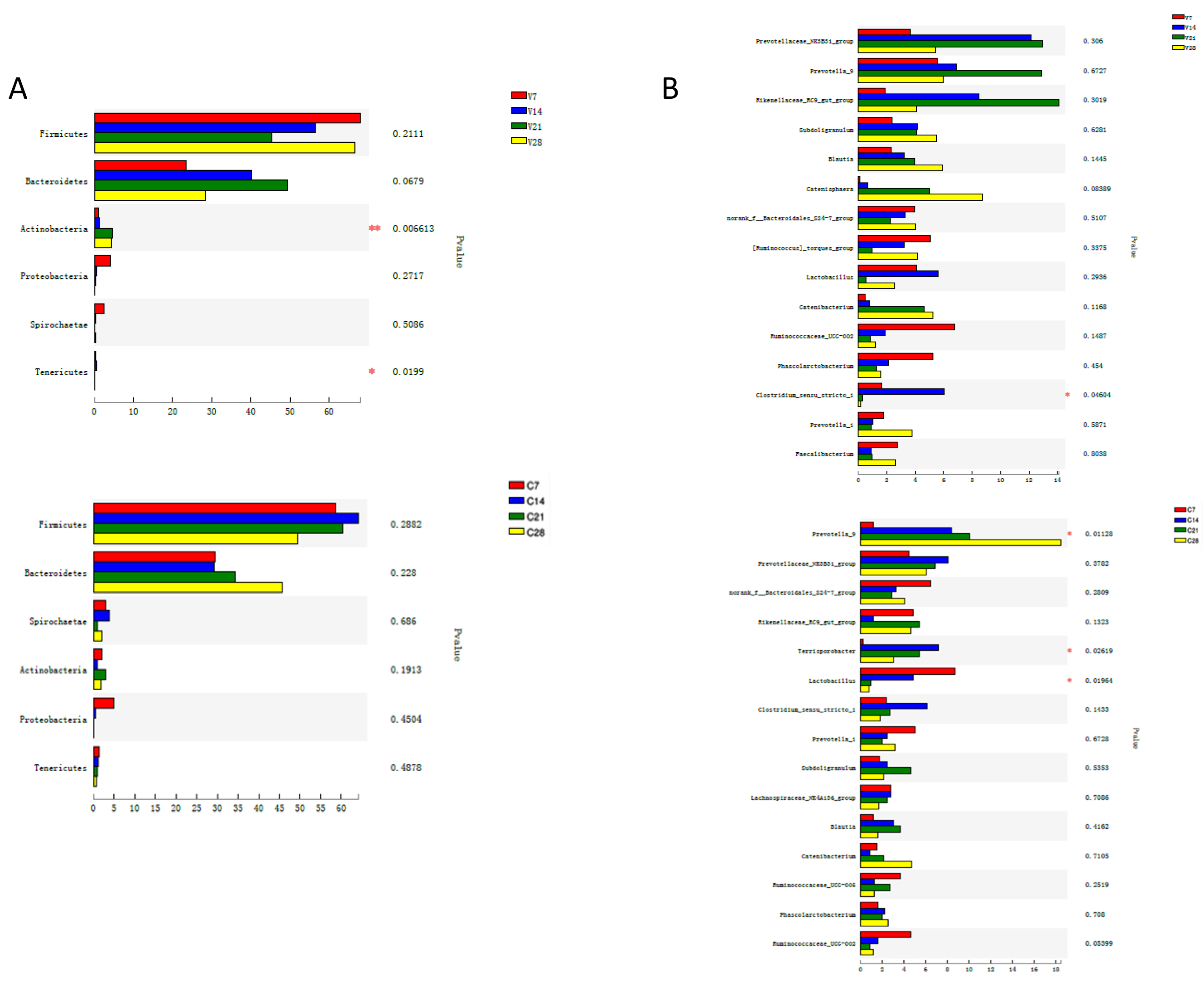

Supplement: FIGURE S3 — The differences in predominant bacterial phyla and genera are illustrated by the Community barplot at 21 dpi in PCV3- (V) and sham-inoculated (C) piglets. Percentage denotes relative abundance of bacterial phylum (A) and genus (B) in each group, with four piglets per group. Statistical analysis was calculated. Values are expressed as mean ± SD, with four piglets per group. ∗P < 0.05, ∗∗P < 0.01. [file Image_3.TIF]
